# Density Functional Theory Study of Optical and Electronic Properties of (TiO_2_)_n=5,8,68_ Clusters for Application in Solar Cells

**DOI:** 10.3390/molecules26040955

**Published:** 2021-02-11

**Authors:** Ife Fortunate Elegbeleye, Nnditshedzeni Eric Maluta, Rapela Regina Maphanga

**Affiliations:** 1 Department of Physics, University of Venda, Thohoyandou 0950, South Africa; ifelove778@gmail.com; 2 National Institute for Theoretical Physics (NITheP), Gauteng 2000, South Africa; rmaphanga@csir.co.za; 3 Next Generation Enterprises and Institutions, Council for Science and Industrial Research, P.O. Box 395, Pretoria 0001, South Africa

**Keywords:** density functional theory, titanium dioxide, optical properties, electronic properties, solar cells

## Abstract

A range of solution-processed organic and hybrid organic−inorganic solar cells, such as dye-sensitized and bulk heterojunction organic solar cells have been intensely developed recently. TiO_2_ is widely employed as electron transporting material in nanostructured TiO_2_ perovskite-sensitized solar cells and semiconductor in dye-sensitized solar cells. Understanding the optical and electronic mechanisms that govern charge separation, transport and recombination in these devices will enhance their current conversion efficiencies under illumination to sunlight. In this work, density functional theory with Perdew-Burke Ernzerhof (PBE) functional approach was used to explore the optical and electronic properties of three modeled TiO_2_ brookite clusters, (TiO_2_)_n=5,8,68_. The simulated optical absorption spectra for (TiO_2_)_5_ and (TiO_2_)_8_ clusters show excitation around 200–400 nm, with (TiO_2_)_8_ cluster showing higher absorbance than the corresponding (TiO_2_)_5_ cluster. The density of states and the projected density of states of the clusters were computed using Grid-base Projector Augmented Wave (GPAW) and PBE exchange correlation functional in a bid to further understand their electronic structure. The density of states spectra reveal surface valence and conduction bands separated by a band gap of 1.10, 2.31, and 1.37 eV for (TiO_2_)_5_, (TiO_2_)_8_, and (TiO_2_)_68_ clusters, respectively. Adsorption of croconate dyes onto the cluster shifted the absorption peaks to higher wavelengths.

## 1. Introduction

In recent years, there has been alarming increase in energy demand across the globe [1,2,3]; fossil fuels (coal, oil and natural gas) constitute a larger percentage of energy being produced and utilized in meeting global energy demands around the world. Fossil fuels need to be replaced by cleaner and cheaper renewable energy sources for compelling environmental and economic challenges in the 21st century. Solar energy with its unlimited quantity is expected to be one of the most promising alternative energy sources in the future [4,5,6]. Various solar energy devices have been widely utilized for photovoltaic (PV) applications; these include silicon [7,8,9], copper indium gallium selenium [10,11,12], cadmium telluride [13,14], concentrating PV [15,16,17], and organic solar cells such as dye sensitized solar cells [18,19]. Innovation that includes multiple excitonic concepts, band gap engineering, introduction of quantum dots and nanotechnology particles having higher efficiencies is growing in scientific research and in laboratory scale production to improve the absorption level and maximize the efficiency of these solar cells [20,21]. The major challenges limiting the widespread utilization of solar energy include cost, toxicity and efficiency of photovoltaic cells [22,23]. Most PVs currently being manufactured and used worldwide are made of solar grade silicon (Si) but widespread use of silicon-based PV technology is limited by the high cost of silicon at USD 30/kg [24,25]. Devices with low manufacturing cost and high efficiency are therefore a necessity for sunlight capture and light-to-energy conversion [4]. Dye sensitized solar cells (DSSCs) on the other hand offers a low cost and promising high efficiency alternative to the silicon-based PV [18,19,25].

Titanium dioxide, also known as titania, belongs to the class of transitions metal oxides [26]. TiO_2_ has attracted a wide range of interests in the research community owing to its proven ability for many applications in the medical field [27,28], environmental protection [29,30,31], renewable energy [32], etc. TiO_2_ is widely employed in solar energy devices, typically used as electron transporting material in nanostructured TiO_2_ perovskite-sensitized solar cells and semiconductor in dye sensitized solar cell architecture [18,19,33,34]. The typical DSSC employs a photoanode, which comprises organic dye that is chemically anchored onto a nanostructured TiO_2_ semiconductor oxide, a catalyst coated transparent conducting oxide and a redox couple triiodide electrolyte (I^−^/I_3_^−^) to generate electricity from sunlight [35,36]. The dye plays a vital role in generation of electric current because the basic function of light absorption, charge injection and regeneration of electrons of the oxidized electrolyte is performed by the dye molecules [37]. The dye absorbs photons of solar radiation and transfers photo generated electrons into the band gap of the semiconductor. The absorption of photons from sunlight in dye sensitized solar cell occurs by mechanism of photocatalysis.

TiO_2_ thin films are extensively studied owing to their fascinating chemical, electrical and optical properties. The interest in TiO_2_ as semiconductor material for dye sensitized solar cell is motivated by its low cost, nontoxicity and good stability upon illumination [38,39,40,41]. Despite the existence of various semiconductor metal oxides with wide bandgap such as SnO_2_ and ZnO, TiO_2_ thin film is one of the most investigated materials as photo-electrode for DSSCs application owing to their lower susceptibility to photo degradation under illumination to sunlight. Additionally, the efficiency of DSSCs constructed with TiO_2_ electrodes yield the highest values of short circuit current (I_sc_), open circuit voltage (V_oc_), conversion efficiency(ŋ) as well as incident photon conversion efficiency (IPCE) [42].

TiO_2_ exists in three major polymorphs (anatase, rutile and brookite). Anatase and rutile phases of TiO_2_ can be synthesized by sol-gel method but brookite is often observed as a by-product of precipitation in an acidic medium at low temperature. Pure brookite without rutile or anatase is rather difficult to synthesize and hence there are limited studies on it; its photocatalytic properties have not been studied much [43,44]. TiO_2_ has a wide band gap of about 3.2–3.4 eV, which often limits its photocatalytic application. To improve the efficiency of TiO_2_ under light illumination, additives can be incorporated into TiO_2_ so that it can be active in the visible and near infra-red region with reduced band gap energy [45]. The incorporation of doping materials (dye molecules or atoms) into TiO_2_ semiconductor could narrow the wide band gap of TiO_2_, which restricts its spectral characteristics to the UV region of the solar spectrum. Optimization of the adsorption of dye molecules on surfaces of TiO_2_ brookite semiconductor can narrow the band gap of TiO_2_ and improve light harvesting efficiency to absorb in the visible and near infra-red region.

A significant number of studies have been done towards the surface modification of TiO_2_ crystals; these include energy band modulation by elemental doping, monodoping, codoping with nonmetals and transition metals, and adsorption of dye molecules on TiO_2_ surfaces. The results showed improved spectral response and enhanced photocatalytic performances of TiO_2_ [46,47]. The adsorption of sensitizing dye molecules to the surface of TiO_2_ to stepwise reduce the band gap can enhance further their activities in the visible and near infra-red region of the solar spectrum. Dyes that have been adsorbed on TiO_2_ are ruthenium complexes [48,49], coumarin dyes [50], acetic dyes [51], phenothiazines dyes [52], cyanidin dyes [53], anthocyanidin and anthocyanin dyes [54]. The adsorption of the aforementioned dyes on TiO_2_ has been greatly exploited and reported in literature [48,50,51,52,53,54,55] but anatase and rutile polymorphs of TiO_2_ have been models for such studies, aimed at improving photocurrent yield and light harvesting in DSSCS. Relatively limited work has been done on the brookite form of TiO_2,_ in contrast to rutile and anatase polymorphs that have been greatly exploited [34,56]. Jeffrey et al. investigated polaron formation in anatase and brookite TiO_2_ using DFT and DFT + U [57]. Puyad et al. reported on the adsorption of croconate dyes on TiO_2_ anatase (101) surfaces [58]. Chitumetalla et al. reported on substituent effects on the croconate dyes in dye sensitized solar cell applications [59]. Elegbeleye et al. reported on DFT studies of ruthenium N3 sensitizers on TiO_2_ brookite cluster [49]. In another study, Elegbeleye et al. reported on the optical and electronic properties of polyenediphenyl-aniline dye, croconate dye and its adsorption on TiO_2_ brookite cluster [60]. One of the fascinating features of croconate dyes is their good bathochromic shift, they can absorb photons in the near infrared region. The croconate dye contains the short oxyallyl cation subgroup as a backbone, which makes it easily synthesizable thereby producing flexible DSSCs with strong light interaction [61]. Additionally, croconate dyes exhibit narrow and intense near infra-red region light absorption peaks and harness light at absorption wavelengths that are higher than 1100 nm [58,59,61].

In this work, DFT with PBE functional computational approach was used through various computational software within an atomic simulation environment to explore the optical and electronic properties of three modeled TiO_2_ brookite clusters, namely Ti_5_O_10_, Ti_8_O_16_ and Ti_68_O_136_ for application in organic and hybrid organic–inorganic solar cells. We subsequently investigated the interaction of visible and near infra-red light absorbing croconate dye molecules with the clusters. We report on the adsorption energies, UV/Vis adsorption spectra, total density of states, projected density of states, and the isodensity surfaces of the key molecular orbital involved in photoexcitation.

## 2. Results and Discussion

### 2.1. Optical Properties of (TiO_2_)_5_ and (TiO_2_)_8_ Brookite Clusters

**Figure 1:**
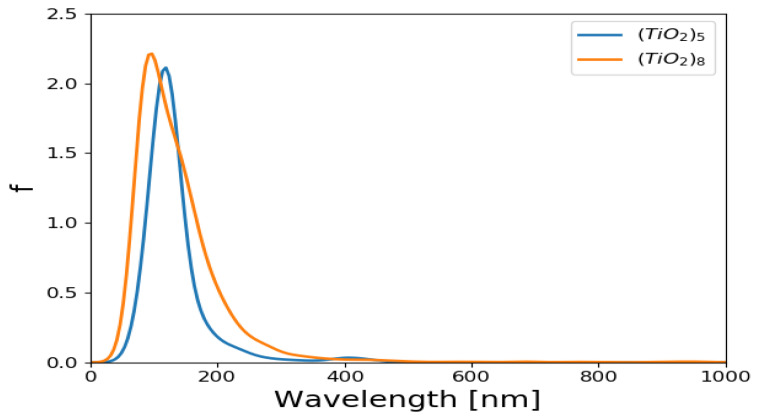
UV/Vis absorption spectrum for (TiO_2_)_5_ and (TiO_2_)_8_ brookite cluster. The wavelengths were folded by Gaussians of $e_{min}=100$, $e_{max}=1200$ nm width. The *y*-axis is folded oscillator strength (1/nm).

The absorption spectra of (TiO_2_)_5_ and (TiO_2_)_8_ brookite clusters were simulated in vacuum using TD-DFT method [62] in which the PBE exchange correlation functional was used for the TD-DFT calculations. Absorption spectrum of (TiO_2_)_68_ was not computed owing to its periodicity. The UV/Vis absorption spectra for (TiO_2_)_5_ brookite nanoclusters and (TiO_2_)_8_ brookite clusters are presented in Figure 1. The oscillator strengths were folded by Gaussians of $e_{min}=100$, $e_{max}=1200$ nm width. The *y*-axis is “folded oscillator strength [1/nm].

The absorption spectra of (TiO_2_)_5_ and (TiO_2_)_8_ complex presented were computed using GPAW and TD-DFT [62,63] with the PBE exchange correlation functional. The spectra show absorption in the UV region of the solar spectrum, with the absorption peaks predominantly situated around 200 nm in the UV region. The (TiO_2_)_5_ also shows a slight absorption peak at around 400 nm, suggesting the absorption peak at higher wavelength than (TiO_2_)_8_. Maximum peak height indicates that the higher absorbance is notable in (TiO_2_)_8_ brookite absorption spectra than in the (TiO_2_)_5_ absorption spectra. The results of the absorption spectra of (TiO_2_)_5_ and (TiO_2_)_8_ generally agrees with findings from literature in the sense that TiO_2_ is majorly sensitive in the UV region of the solar spectrum owing to its wide band gap (3.0–3.2 eV) [64,65].

### 2.2. Electronic Properties of (TiO_2_)_n_ (n = 5, 8, 68) Brookite Clusters

**Figure 2:**
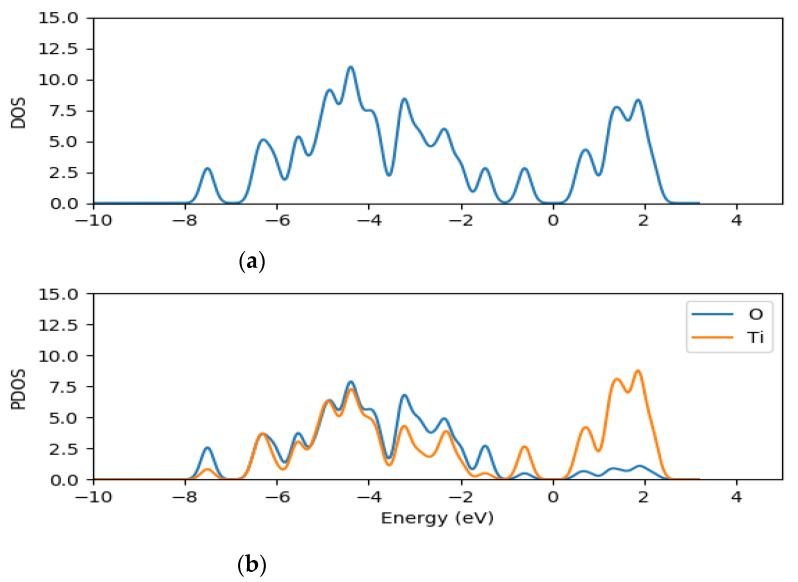
TDOS (**a**) and the projected DOS (**b**) for (TiO_2_)_5_ nanocluster with the orange line representing titanium atom contributions and blue line representing oxygen contributions for PDOS.

**Figure 3:**
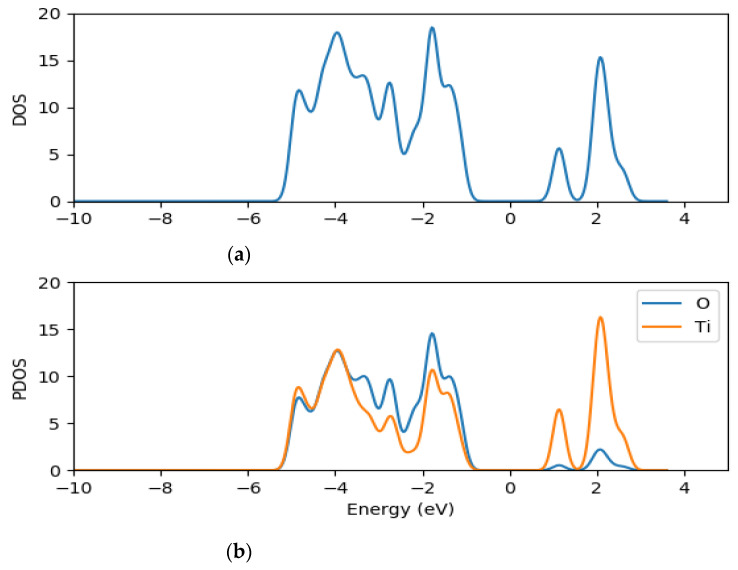
TDOS (**a**) and the projected DOS (**b**) for (TiO_2_)_8_ nanocluster with the orange line representing titanium atom contributions and the blue line representing oxygen contributions for PDOS.

**Figure 4:**
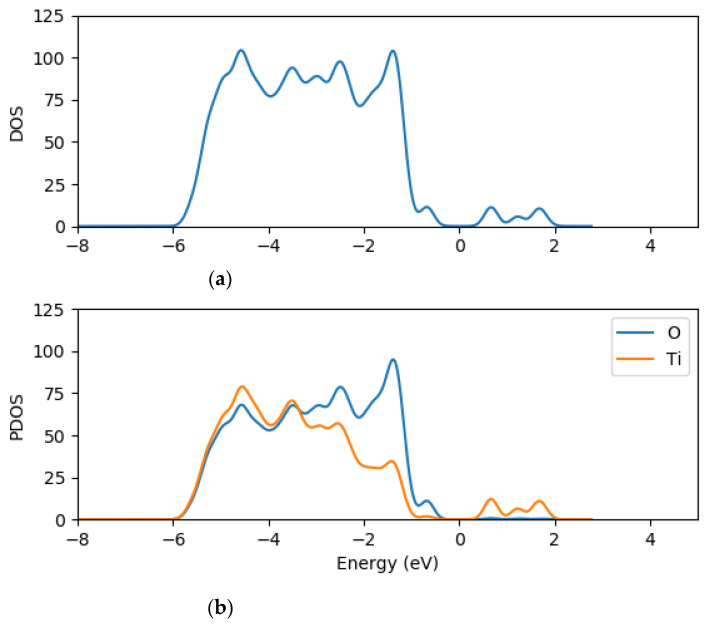
TDOS (**a**) and the projected DOS (**b**) for (TiO_2_)_68_ nanocluster with the orange line representing titanium atom contributions and the blue line representing oxygen contributions for PDOS.

The density of states and the projected density of states of (TiO_2_)_5_, (TiO_2_)_8_ and (TiO_2_)_68_ nanoclusters were computed using GPAW [63] and PBE exchange correction functional [66] in order to understand further the electronic structure of the clusters. The total density of states (TDOS) and projected density of states (PDOS) of (TiO_2_)_5_, (TiO_2_)_8_ and (TiO_2_)_68_ are presented in Figure 2, Figure 3 and Figure 4, respectively.

The density of states (DOS) is composed of the surface valence and conduction bands separated by a wide band gap. The PDOS results for the clusters show that both the oxygen and titanium atomic orbitals contributes to the valence states, with the oxygen 2*p* atomic orbitals contributing mostly to the highest occupied valence band (VB) state, whereas the lowest unoccupied state of the conduction band is mainly dominated by the contributions of titanium 3*d* atomic orbitals as illustrated in Figure 2, Figure 3 and Figure 4. The key contribution in the conduction band comes from the titanium orbitals, especially the *d* and *p* orbitals, while contributions from oxygen atoms are minimal.

### 2.3. Structural Models

The structural model for brookite polymorph of TiO_2_ investigated in this work is shown in the supplementary information (Figure S1). The structures of the TiO_2_ brookite semiconductor material employed in this study was used to generate models of the three TiO_2_ clusters. Brookite TiO_2_ has an orthorhombic crystalline structure with eight formula units in the orthorhombic cell.

The optimized molecular geometries of the croconate dyes CR1 and CR2 considered for this study are presented in the supplementary information (Figure S2). The optimized geometry parameters, i.e., bond lengths and bond angles are presented in Table 1. The O6–C3 bond length of the oxyallyl moiety in CR1 is 1.236 Å; this is longer than the bond length of C5–O7/C1–C8 which is 1.220 Å. This was also the case for the bond length of O6–C3 (1.243 Å) and C5–O9/C1–O10 (1.238 Å) in CR2 and suggests a more single bond character of the oxyallyl compared to C5–O7/C1–C8 and C5–O9/C1–O10. Other findings include values of 1.299 Å O6–C3 bond length for the oxyallyl moiety and 1.213 Å for C5–O7/C1–C8 in CR1, and O6–C3 (1.216 Å) and C5–O9/C1–O10 (1.208 Å) in CR2. Our findings compare favorably with those obtained by others [58,59].

CR1 contains electron donating methyl group (CH_3_), which is an alkyl derived from methane, with one carbon atom bonded to three hydrogen atoms while CR2 contains an electron withdrawing carboxyl group (–COOH), which is an organic compound situated in the carboxylic acid, one carbon atom bonded to two oxygen atoms and one hydrogen atom.

### 2.4. Adsorption of Croconate Dyes on (TiO_2_)_5,_ (TiO_2_)_8_ and (TiO_2_)_68_ Clusters

The croconate dyes CR1 and CR2 are adsorbed onto the surface of (TiO_2_)_5,_ (TiO_2_)_8_ and (TiO_2_)_68_ brookite clusters through the diketo group as presented in Figures S4a,b–S6a,b. The optical properties and electron injection efficiency of two croconate dyes, coded CR1 and CR2 adsorbed on (TiO_2_)_5,_ (TiO_2_)_8_ and (TiO_2_)_68_ brookite clusters are investigated in this section. A fascinating feature about croconate dyes is their good solvatochromic shift, they can absorb photons in the near infrared region [61]. Croconate dyes contained a short oxyallyl subgroup as a backbone, which makes them easy to synthesize and thereby producing DSSCs that are flexible and interact strongly with light [61]. Additionally, croconate dyes exhibit narrow and intense absorption bands in the near infrared (IR) region of the solar spectrum [52,59]. The bidentate bridging (BB) adsorption mode in which each of the oxygen of the keto moiety binds to a three-fold coordinated [44,50] titanium atom was adopted because it was previously reported to be more energetically favorable [50,52]. All dye-TiO_2_ complexes were relaxed upon adsorption.

### 2.5. Adsorption Energy of Croconate Dyes Adsorbed on (TiO_2_)_n_ n = 5, 8, 68 Brookite Complex

The computed adsorption energies are presented in Table 2. The adsorption energy of CR1-(TiO_2_)_5_ is 3.93 eV, CR2-(TiO_2_)_5_ is 5.53 eV, CR1-(TiO_2_)_8_ is 0.75 eV, CR2-(TiO_2_)_8_ is 0.68 eV, CR-(TiO_2_)_68_ is 4.74 eV, and CR2-(TiO_2_)_68_ is 4.95 eV.

The positive adsorption energies denote the binding ability of the dye molecules to the surface of TiO_2_ nanocluster [58]. The results show that the dye with the electron donating methyl (CR1) binds more strongly to the surface of (TiO_2_)_8_ brookite cluster than the one with the electron withdrawing moiety (CR2). However, the dye with the electron withdrawing moiety (CR2) binds more strongly to (TiO_2_)_5_ and (TiO_2_)_68_ brookite cluster than the dye with the electron donating methyl (CR1). The results suggest that the dye molecules react differently to different surfaces and sizes of TiO_2_ brookite clusters.

### 2.6. Absorption Spectrum of CR1 and CR2 Dyes Adsorbed on (TiO_2_)_n_ n = 5, 8 Brookite Clusters

**Figure 5:**
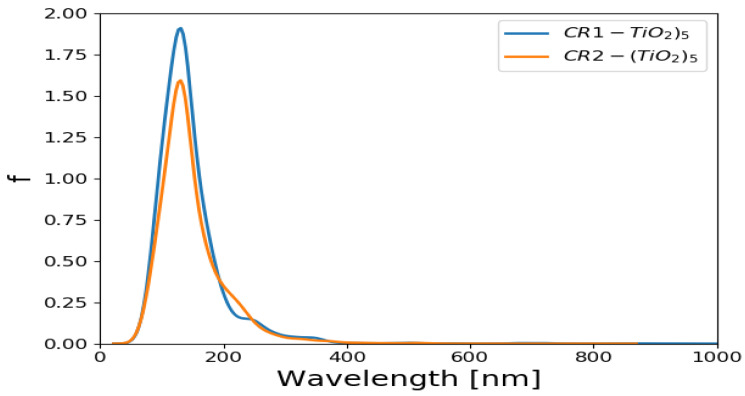
Simulated UV/Vis absorption spectrum of CR1 and CR2 absorbed on (TiO_2_)_5_ brookite cluster. The oscillator strengths were folded by Gaussians of *e_min_* = 100, *e_max_* = 1200 nm width. The *y*-axis is folded oscillator strength (1/nm).

**Figure 6:**
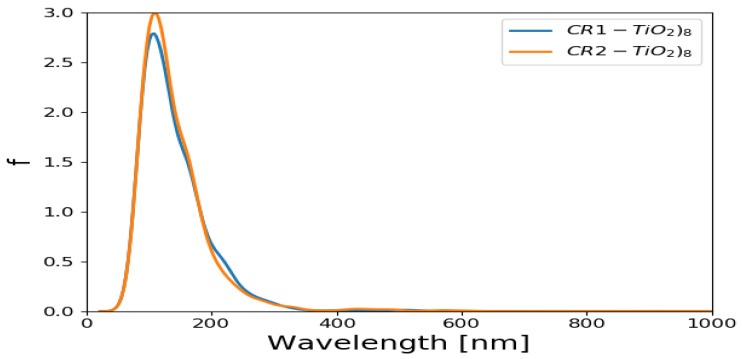
Simulated UV/Vis absorption spectrum of CR1 and CR2 absorbed on (TiO_2_)_8_ brookite. The oscillator strengths were folded by Gaussians of *e_min_* = 100, *e_max_* = 1200 nm width. The *y*-axis is folded oscillator strength (1/nm).

The optical spectra of CR1 and CR2 dyes adsorbed on the (TiO_2_)_5_ are presented in Figure 5 and Figure 6. The calculated optical spectra evidently show that the absorption maxima of the cluster with dye molecules have shifted to higher wavelength compared to the optical spectrum of a clean (TiO_2_)_5_ cluster. The absorption maxima of (TiO_2_)_5_ brookite cluster, which was located around 200 nm has now shifted to higher wavelength around 700–900 nm as shown in Figure 5. The optical spectra of CR1 and CR2 dye molecules adsorbed on the (TiO_2_)_8_ are presented in Figure 6. These were compared with the spectrum for the optical spectra of (TiO_2_)_8_ cluster and it is evident that the absorption maxima with the dyes have shifted to higher wavelength. The absorption maxima of (TiO_2_)_8_ brookite cluster that was located around 200 nm has now shifted to higher wavelength around 600–900 nm. In both cases, the CR1 and CR2 dye molecules show a bathochromic shift upon adsorption of the dyes on TiO_2_ cluster. The bathochromic shift observed after adsorption of CR1 and CR2 dye molecules on TiO_2_ brookite cluster suggests good optical properties of the dye molecules and corroborates with reports from literature that the adsorption of dye molecules on TiO_2_ improves its optical response and helps overcome its limited spectral sensitivity in the UV region, thereby improving its photocatalytic properties and overall DSSCs device efficiencies [65].

### 2.7. Isodensity Surfaces of the Croconate Dyes Adsorbed on (TiO_2_)_n,_ n = 5, 8, 68 Brookite Clusters

**Figure 7:**
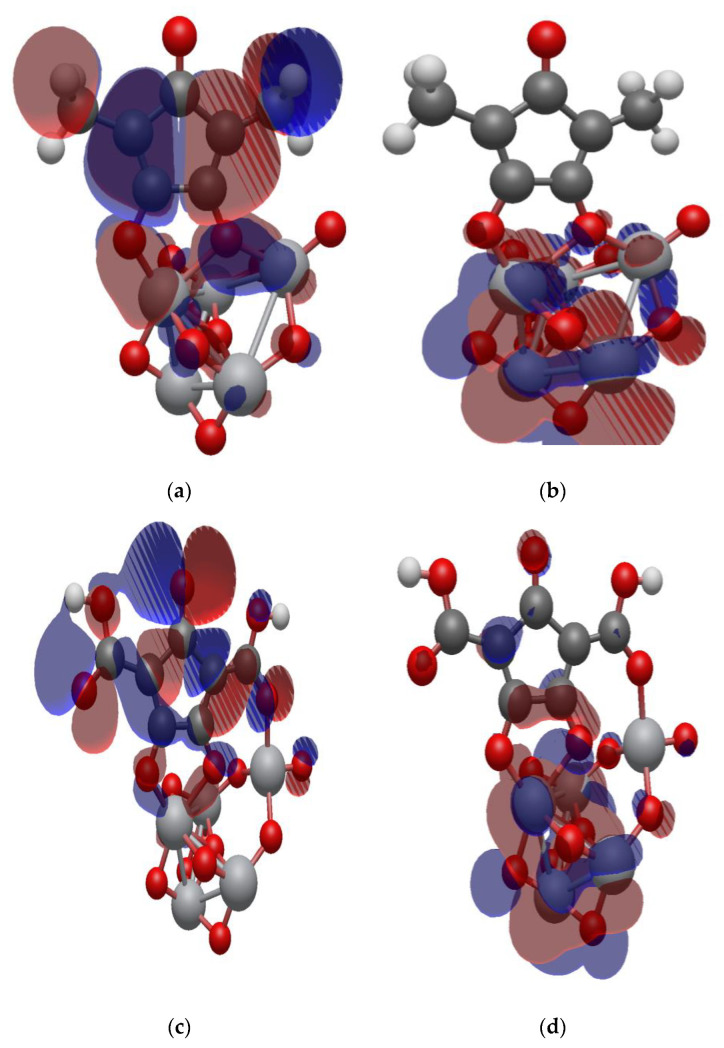
Isodensity surfaces of the molecular orbitals of (**a**) HOMO of CR1-(TiO_2_)_5_ brookite cluster, (**b**) LUMO of CR1-(TiO_2_)_5_ brookite cluster, (**c**) HOMO of CR2-(TiO_2_)_5_ brookite cluster, (**d**) LUMO of CR2-(TiO_2_)_5_ brookite cluster.

**Figure 8:**
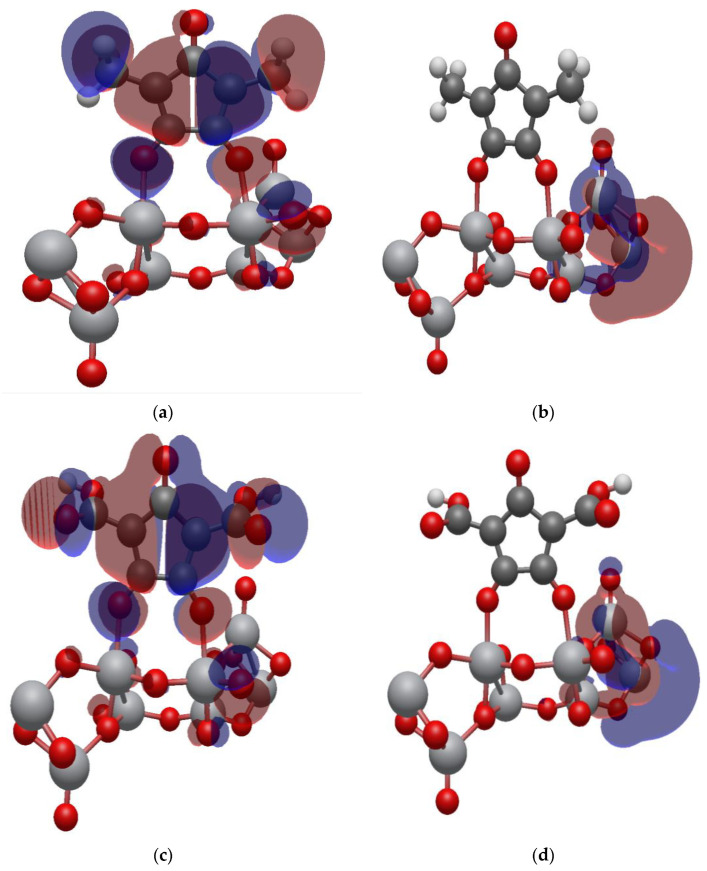
Isodensity surfaces of the molecular orbitals of (**a**) HOMO of CR1-(TiO_2_)_8_ brookite cluster, (**b**) LUMO of CR1-(TiO_2_)_8_ brookite cluster, (**c**) HOMO of CR2-(TiO_2_)_8_ brookite cluster, (**d**) LUMO of CR2-(TiO_2_)_8_ brookite cluster.

**Figure 9:**
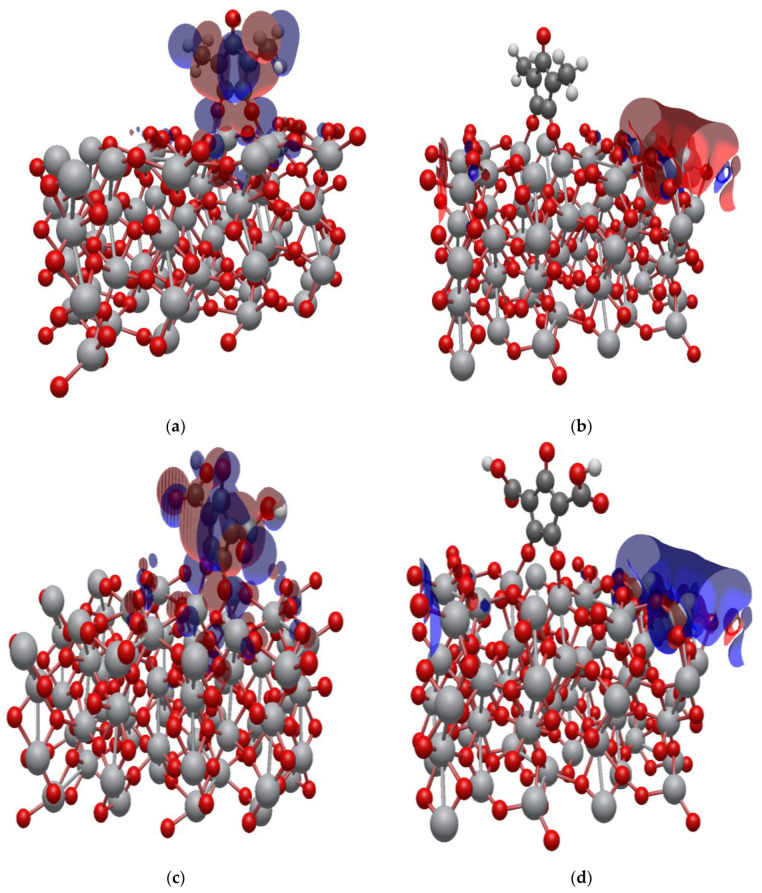
Isodensity surfaces of the molecular orbitals of (**a**) HOMO of CR1-(TiO_2_)_68_ brookite cluster, (**b**) LUMO of CR1-(TiO_2_)_68_ brookite cluster, (**c**) HOMO of CR2-(TiO_2_)_68_ brookite cluster, (**d**) LUMO of CR2-(TiO_2_)_68_ brookite cluster.

The isodensity surfaces of the molecular orbital involved in photoexcitation of CR1 and CR2 dye molecules adsorbed on (TiO_2_)_5_, (TiO_2_)_8_ and (TiO_2_)_68_ brookite cluster are presented in Figure 7, Figure 8 and Figure 9, respectively. All the results show that the HOMO is localized on the dye molecule and it is mainly concentrated on the donor moiety where the occupied electronic orbitals are located while the LUMO is localized over the TiO_2_ clusters where the unoccupied electronic states are situated. This suggests good electronic coupling between the occupied excited state of the dye and the unoccupied acceptor levels of the semiconductor conduction band. The localization of the HOMO electronic level on the dye molecules and the LUMO electronic level on the TiO_2_ clusters implies efficient separation of charge upon adsorption and electron injection from the dye excited state into the TiO_2_ semiconductor conduction band.

### 2.8. Electronic Properties of CR1 and CR2 dye Molecules Adsorbed on TiO_2_ Clusters

**Figure 10:**
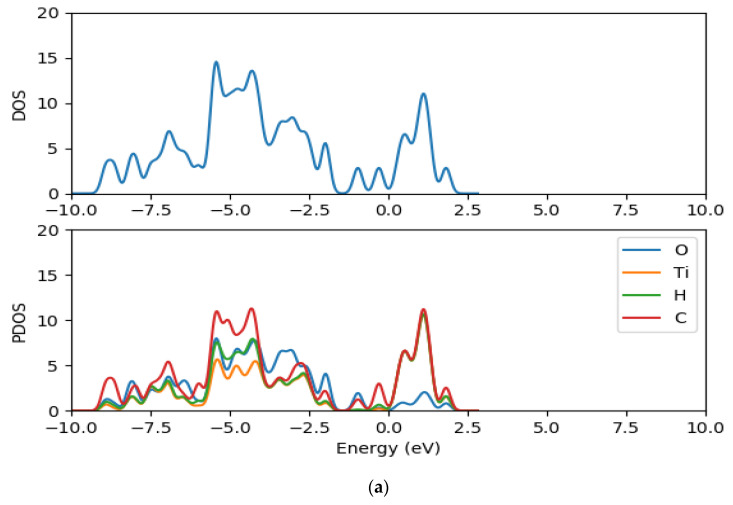

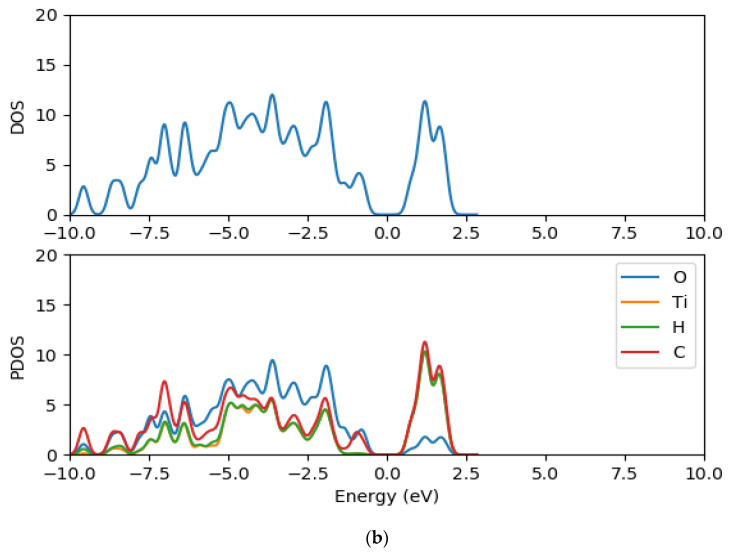
Total density of states and projected density of state spectra of croconate dyes adsorbed on (TiO_2_)_5_ nanocluster (**a**) CR1-(TiO_2_)_5_ DOS and PDOS (**b**) CR2-(TiO_2_)_5_ DOS and PDOS.

**Figure 11:**
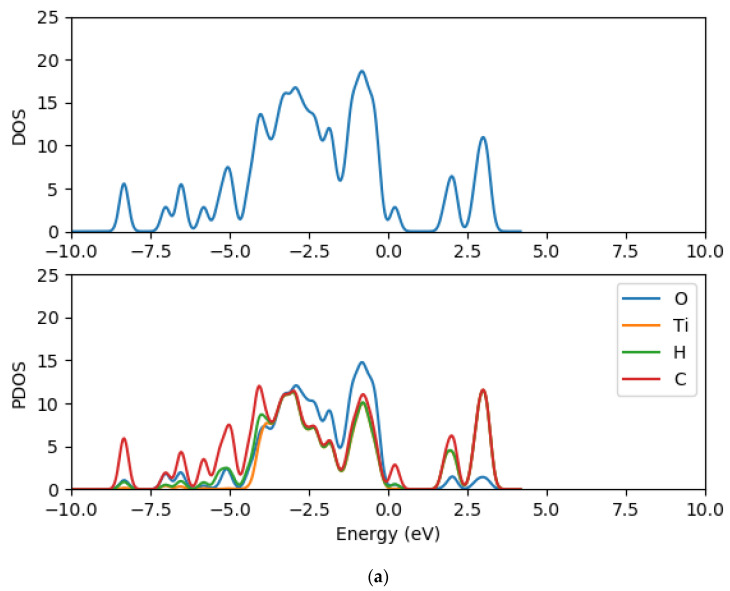

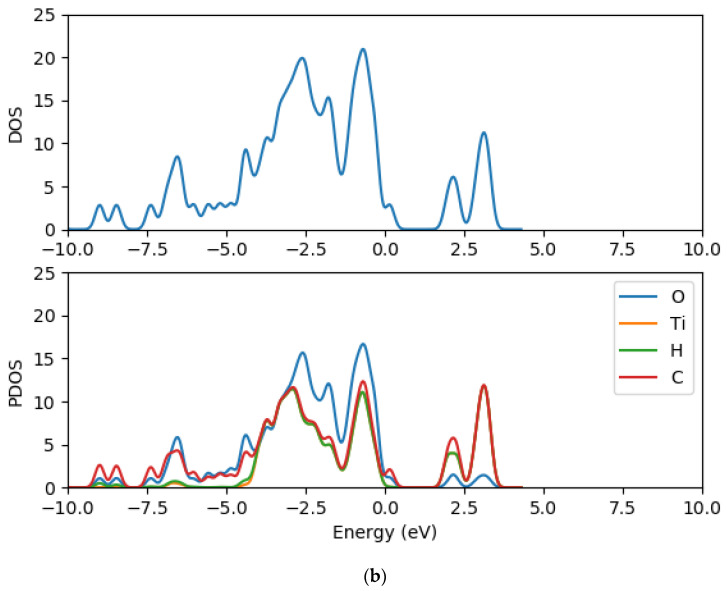
Total density of states and projected density of state spectra of croconate dyes adsorbed on (TiO_2_)_8_ nanocluster (**a**) CR1-(TiO_2_)_8_ DOS and PDOS (**b**) CR2-(TiO_2_)_8_ DOS and PDOS.

**Figure 12:**
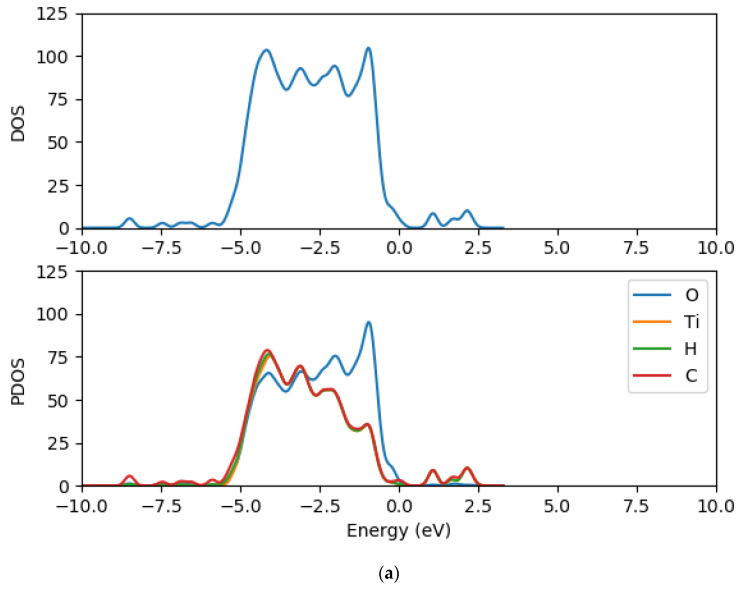

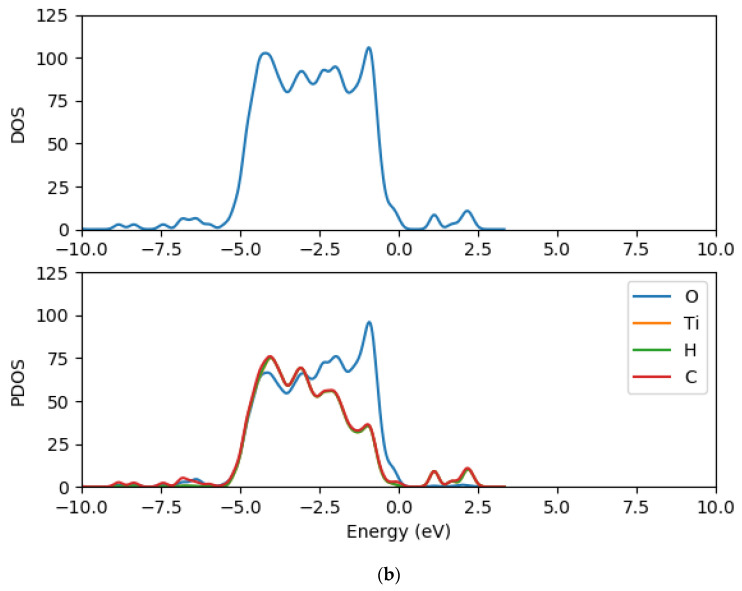
Total density of states and projected density of state spectra of croconate dyes adsorbed on (TiO_2_)_8_ nanocluster (**a**) CR1-(TiO_2_)_8_ DOS and PDOS (**b**) CR2-(TiO_2_)_8_ DOS and PDOS.

In order to understand further the electronic structure of CR1 and CR2 dye molecules adsorbed on TiO_2_ clusters, the density of states and the projected density of states of CR1 and CR2 dye molecules adsorbed on (TiO_2_)_5_, (TiO_2_)_8_ and (TiO_2_)_68_ were computed using GPAW and PBE exchange correction functional. The TDOS and PDOS spectra are presented in Figure 10, Figure 11 and Figure 12 for dyes-(TiO_2_)_5_, dyes-(TiO_2_)_8_ and dyes-(TiO_2_)_68_, respectively. The DOS is composed of the surface valence and conduction bands separated by a wide band gap. The density of states for the clean (TiO_2_)_5_, (TiO_2_)_8_ and (TiO_2_)_68_ clusters before the dyes were adsorbed were presented previously in Figure 7, Figure 8 and Figure 9, respectively. When comparing the DOS spectrum for the TiO_2_ cluster alone and the DOS spectrum of croconate dyes adsorbed on TiO_2_ clusters, it is observed that the adsorption of the CR1 and CR2 dye molecules results in new occupied molecular orbitals introduced to the band gap of the TiO_2_ clusters upon the absorption of the dye molecules as seen in Figure 10, Figure 11 and Figure 12 this is as a result of the contribution of the dye molecular states in the band gap of TiO_2_. Comparing between the DOS spectra of all the clean (TiO_2_)_5_, (TiO_2_)_8_ and (TiO_2_)_68_ clusters and the DOS spectra of the dyes adsorbed on the clusters, it is clear that the adsorption of the dyes on TiO_2_ clusters results in a shift of the conduction band edge of TiO_2_ to higher energy levels, and consequently narrowing of the band gap between the occupied valence states and the unoccupied conduction band. Additionally, upon adsorption of the dye molecules on the TiO_2_ clusters, the DOS results reveal that the process introduces new occupied electronic orbitals between the two states where there was a broad band gap initially and in the conduction band of the TiO_2_ clusters.

The PDOS spectra of the dyes-(TiO_2_)_5_, dyes-(TiO_2_)_8_ and dyes-(TiO_2_)_68_ show the contribution of the atomic orbitals in the occupied states and unoccupied states. The PDOS results for the clean clusters showed that both the oxygen and titanium atomic orbitals contributes to the valence states, the oxygen 2*p* atomic orbitals contributes mostly to the highest occupied valence band state, whereas the lowest unoccupied state of the conduction band is mainly dominated by the contributions of titanium 3*d* atomic orbitals. The valence band is dominated by the *p* atomic orbitals of oxygen with a little contribution from the *p* atomic orbitals of titanium. The major contribution in the conduction band comes from the titanium orbitals, especially the *d* and *p* orbitals. The PDOS spectra of all the dyes-(TiO_2_)_5_, dyes-(TiO_2_)_8_ and dyes-(TiO_2_)_68_ complexes show major contributions from the 3*d* orbitals of titanium, 2*p* orbitals of carbon, 2*p* orbitals oxygen, *s* orbitals of hydrogen to the valence states. Carbon *p* orbitals contribute majorly to the conduction band.

## 3. Materials and Methods

### Computational Details

The bulk structure of brookite TiO_2_ that was used throughout the study was imported from Materials Studio Accelrys and optimized using CASTEP module, to obtain the ground state structure of the TiO_2_ brookite semiconductor. The convergence energy cut-off and k-points were set as 650 eV and 4 × 7 × 7, respectively, and bulk properties were previously reported in [49]. The three brookite clusters with stoichiometry (TiO_2_)_n_ where *n* = 5, 8 and 68 were modeled from the optimized ground state bulk structure of TiO_2_ brookite. (TiO_2_)_5_ brookite cluster is a nanostructured form of brookite TiO_2_ modeled from the bulk structure with dimension repeated in x, y and z directions. (TiO_2_)_8_ brookite cluster with the stoichiometry of (TiO_2_)_8_ is modeled from the bulk structure of brookite, while (TiO_2_)_68_ is a supercell which was created from the repetition of the optimized TiO_2_ brookite bulk structure unit cell in repeated by 2 × 2 × 2 Å in three dimensions. The trend for TiO_2_ is (TiO_2_)n with Ti and O in the ratio 1:2, respectively. The clusters were then exported to (GPAW) [63] software via crystallographic information format (.cif) and were visualized using Avogadro software [67] for further computational analysis and calculations.

All DFT calculations were performed within an atomic simulation environment (ASE) with GPAW software while the structures were visualized with Avogadro. GPAW is a Python based program-package formulated with density-functional theory (DFT) combined with the grid space projector-augmented wave (GPAW). The three TiO_2_ brookite clusters that were generated are presented in Figure S3a–c (see supplementary). Figure S3a comprises (TiO_2_)_5_ brookite nanocluster with five titanium and 10 oxygen atoms cleaved from brookite bulk structure as described in the previous section. Figure S3b depicts brookite (TiO_2_)_8_ comprising eight titanium and 16 oxygen atoms whereas Figure S3c reveals a periodic brookite (TiO_2_)_68_ supercell 2 × 2 × 2 Å comprising 68 titanium atoms and 136 oxygen atoms.

All the structures were relaxed in vacuum using GPAW with the PBE. The exchange correlation energy was approximated within the generalized gradient approximation PBE [66] whereby the final lowest energy structures were determined. The PBE exchange functional was found to reproduce the experimental band gaps of bulk brookite TiO_2_ as well as the main features of oxygen vacancies in rutile and anatase polymorphs. GPAW implements the projector-augmented wave method with the smooth wave-functions and electron density represented on real space grids. The structures were considered to have converged when the maximum forces that were acting on all the atoms were about 0.05 N. The periodic boundary conditions were implemented on the supercell during the relaxation and was set to none for the nonperiodic brookite cluster models. The atoms of the cluster were reoriented during the relaxation until the ground state structure was reached, where they became stable and the forces converged to a maximum value of 0.05 N.

The UV/Vis, total density of states (TDOS) and partial density of states (PDOS) of the nanocluster structures were computed using the trajectory files obtained from the relaxed structures. The UV/Vis was calculated in vacuum and the TDOS and PDOS were computed using the PBE functional.

## 4. Conclusions

First principle calculations based on density functional theory (DFT) and time dependent (TD-DFT) were used to investigate the optical and electronic properties of (TiO_2_)_5_, (TiO_2_)_8_ and (TiO_2_)_68_ clusters. We also investigated the adsorption of visible and near infra-red light harvesting croconate dyes onto the three TiO_2_ brookite clusters, namely (TiO_2_)_5_, (TiO_2_)_8_ and (TiO_2_)_68_. Our findings reveal that (TiO_2_)_5_ cluster shows higher wavelength absorption than the corresponding (TiO_2_)_8_ and (TiO_2_)_68_ clusters owing to its miniaturized size. The band gap of (TiO2)_5_ cluster also shows a smaller band gap than the corresponding (TiO_2_)_8_ and (TiO_2_)_68_ clusters, which generally suggests that the nanostructure of TiO_2_ will enhance more charge transport for solar cell applications. The simulated optical absorption spectrum for (TiO_2_)_5_ and (TiO_2_)_8_ cluster shows excitation around 200–400 nm, with (TiO_2_)_8_ cluster showing higher absorbance than the corresponding (TiO_2_)_5_ cluster. The computed density of states and the projected density of states spectra for (TiO_2_)_5_, (TiO_2_)_8_ and (TiO_2_)_68_ clusters reveal surface valence and conduction bands separated by energy band gaps of 1.10, 2.31, and 1.37 eV for (TiO_2_)_5_, (TiO_2_)_8_ and (TiO_2_)_68_ clusters, respectively. The projected density of states spectrum reveals that 2*p* atomic orbitals contribute mostly to the highest occupied valence band state, whereas the lowest unoccupied state of the conduction band is mainly dominated by the contributions of titanium 3*d* atomic orbitals. Our findings showed that the adsorption of croconate dyes on the cluster shifted the absorption spectrum to higher wavelengths. The absorption maxima of the clusters alone located in the UV region was shifted to the visible and near infra-red region of the solar spectrum, making it possible to absorb in the whole spectral region. The isodensity surfaces show that HOMO were more localized on the dye while the LUMO were delocalized on the TiO_2_ semiconductor, depicting that electrons were transferred from the dye excited states to the semiconductor acceptor states. Our findings generally show that the optical and electronic properties of TiO_2_ cluster vary with the size of the cluster.

Findings from this research are of good significance as the theoretical knowledge and the results could be a guide to experiments on dye molecules adsorbed onto brookite polymorph for application as electron transporting material in nanostructured TiO_2_ perovskite-sensitized solar cells and as photoanode in dye sensitized solar cells. One of the major challenges limiting the widespread and outdoor application of perovskite solar cells is UV degradation under long-term exposure to sunlight. The adsorption of dye molecules on TiO_2_ brookite proffer a solution to this problem by shifting absorption peaks to the visible and infrared region. The incorporation of these research findings into perovskite solar cell architecture is expected to enhance the widespread and outdoor application because the absorption peaks are shifted to the UV and visible region which makes TiO_2_ (electron transport material in this class of solar cells) less susceptible to UV degradation. The research also provides significant findings for the fabrication of brookite semiconductor based DSSCs.

## Figures and Tables

**Table 1:** Optimized structural parameters of two model croconate dyes, CR1 and CR2. Bond distance and angle are given in units of Å and (˚), respectively.

Dye	O6–C3 (Å)	C3–C4/ C3–C2 (Å)	C4–R/ C2–R (Å)	C5–O7/C1–O8 C5–O9/C1–O10 (Å)	O6C3C4/ O6C3C2 (°)	C4C3C2 (°)
CR1	1.236	1.459	1.464	1.220	127.3	105.1
CR2	1.243	1.468	1.469	1.238	128.6	102.3

**Table 2:** Adsorption energies of croconate dye molecules absorbed on TiO_2_ complex.

Adsorption Energy of Dyes-(TiO_2_)_n=5,8,68_ (eV)		
Dyes-(TiO_2_)_5_	CR1-(TiO_2_)_5_	3.932
	CR2-(TiO_2_)_5_	5.531
Dyes-(TiO_2_)_8_	CR1-(TiO_2_)_8_	0.751
	CR2-(TiO_2_)_8_	0.682
Dyes-(TiO_2_)_68_	D5-(TiO_2_)_68_	4.743
	D7-(TiO_2_)_68_	4.947

## Data Availability

Not applicable.

## Supplementary Materials

The following are available online, Figure S1: Crystallographic forms of TiO_2_ brookite. Figure S2: (**a**) CR1 (**b**) CR2. The atoms are represented according to following colour scheme: grey balls represent titanium atoms, red balls represent oxygen atoms and white ball represent hydrogen atoms. Figure S3: (**a**) (TiO_2_)_5_ brookite cluster; (**b**) (TiO_2_)_8_ brookite cluster; (**c**) (TiO_2_)_68_ brookite cluster. Figure S4: Croconate dyes absorbed on (TiO_2_)_5_ nanocluster (**a**) CR1-(TiO_2_)_5_ (**b**) CR2-(TiO_2_)_5_. Figure S5: Croconate dyes absorbed on (TiO_2_)_8_ nanocluster (**a**) CR1-(TiO_2_)_8_ (**b**) CR2-(TiO_2_)_8_. Figure S6: Croconate dyes absorbed on (TiO_2_)_68_ nanoclusters (**a**) CR1-(TiO_2_)_8_ (**b**) CR2-(TiO_2_)_68_. Figure S7: Expanded view of simulated UV-Vis spectrum of CR1 and CR2 absorbed on (TiO_2_)_5_ brookite cluster. The os-cillator strengths were folded by Gaussians of *e_min_* = 100, *e_max_* = 1200nm width. The *y*-axis is “folded oscillator strength [1/nm]. Figure S8: Expanded view of simulated UV/Vis absorption spectrum of CR1 and CR2 absorbed on (TiO_2_)_8_ brookite cluster. The oscillator strengths were folded by Gaussians of *e_min_* = 100, *e_max_* = 1200 nm width. The *y*-axis is “folded oscillator strength [1/nm].
