# Supplementary material for: Density Functional Theory Study of Optical and Electronic Properties of (TiO2)n=5,8,68 Clusters for Application in Solar Cells
Source: Molecules. 2021 Feb 11;26(4):955. doi: 10.3390/molecules26040955 (PMC7916945; doi:10.3390/molecules26040955)
Supplement: Supplementary file 1 [file molecules-26-00955-s001.pdf]

molecules

MDPI

Article

Density functional theory study of optical and electronic properties of $(TiO_2)_{n=5,8,68}$ clusters for application in solar cells

Ife Fortunate Elegbeleye[1], Nnditshedzeni Eric Maluta[1,2,] * and Rapela Regina Maphanga[2, 3]

1 Department of Physics, University of Venda, Thohoyandou, South Africa; ifelove778@gmail.com.
2 National Institute for Theoretical Physics (NITheP), Gauteng, South Africa; Eric.Maluta@univen.ac.za
3 Next Generation Enterprises and Institutions, Council for Science and Industrial Research, P.O. Box 395, Pretoria, South Africa; rmaphanga@csir.co.za
* Correspondence: Eric.Maluta@univen.ac.za

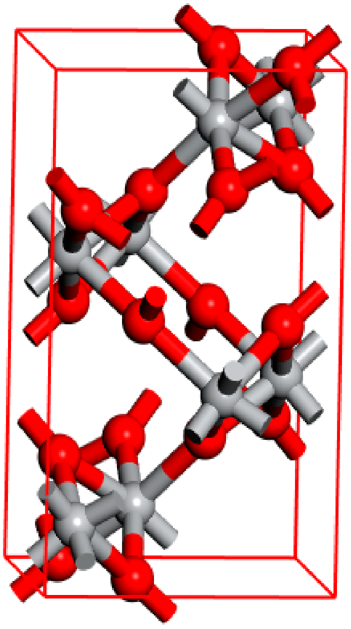

Figure 1. Crystallographic forms of TiO_2 brookite. The atoms are represented according to these colour schemes, grey balls are titanium atoms and red balls are oxygen atoms.

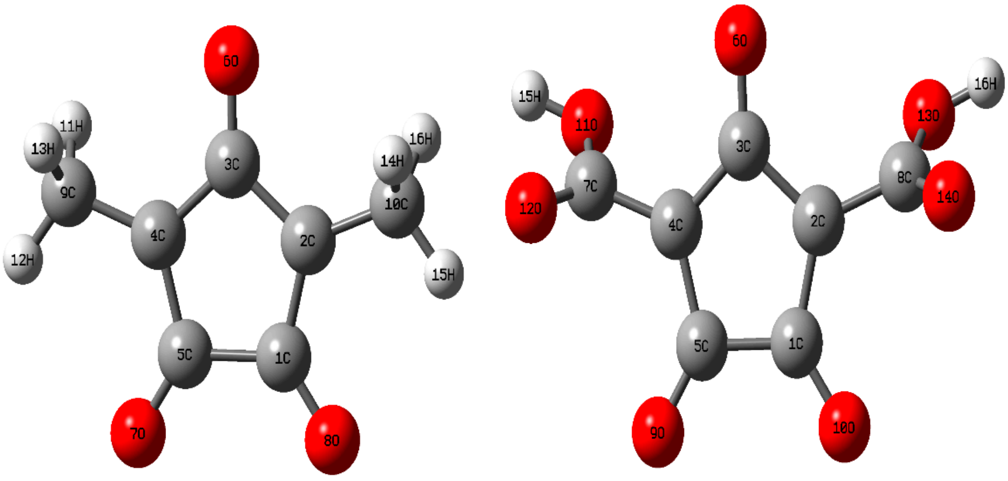

(a) **(b)**

Figure 2. a)-(b) (a) CR1 (b) CR2. The atoms are represented according to following colour scheme: grey balls represent titanium atoms, red balls represent oxygen atoms and white ball represent hydrogen atoms.

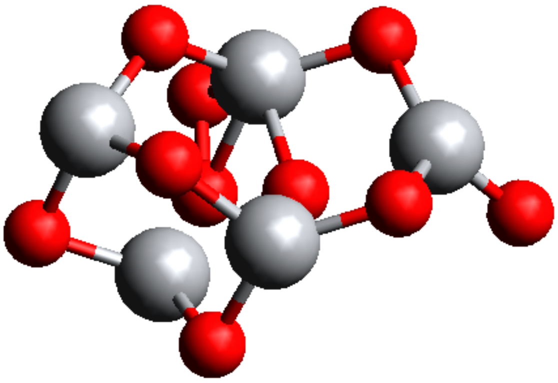

Figure 3. a). $(TiO_2)_5$ brookite cluster.

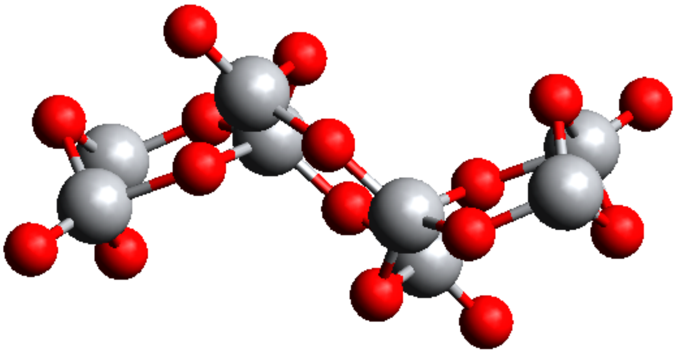

Figure 3. b). $(TiO_2)_8$ brookite cluster.

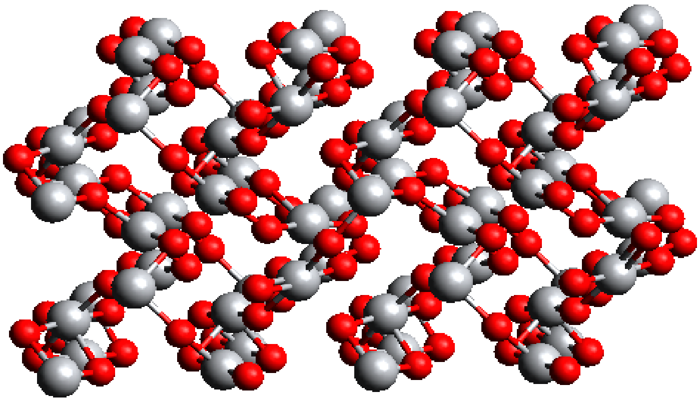

Figure 3. c). $(TiO_2)_{68}$ brookite cluster.

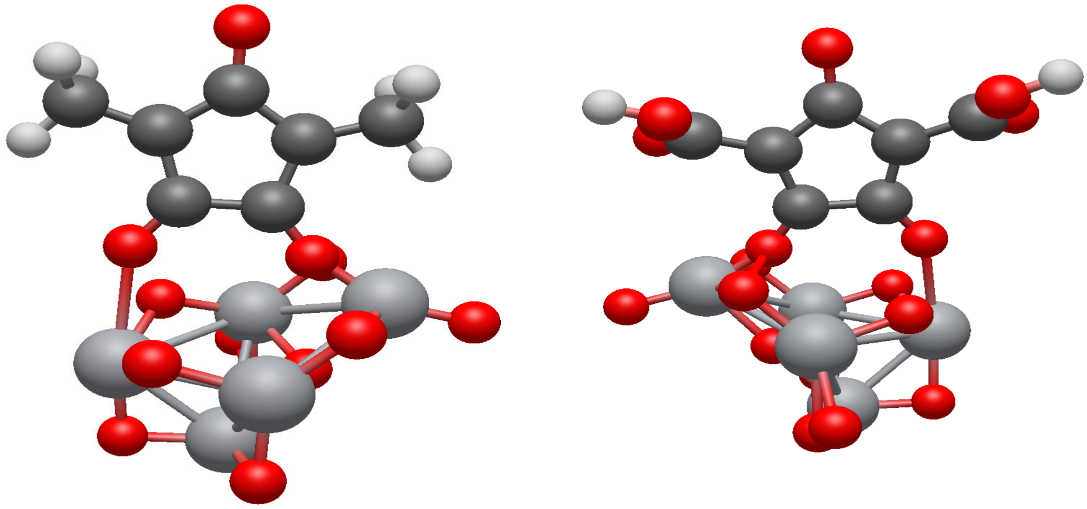

(a) **(b)**

Figure 4. Croconate dyes absorbed on $(TiO_2)_5$ nanocluster (a) CR1@ $(TiO_2)_5$ (b) CR2@ $(TiO_2)_5$.

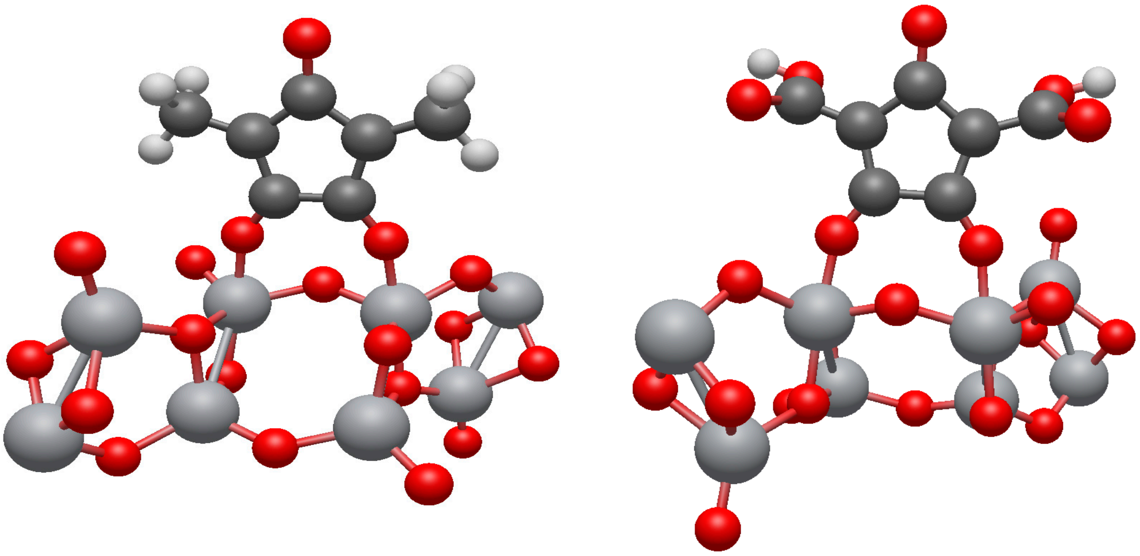

(a) **(b)**

Figure 5. Croconate dyes absorbed on $(TiO_2)_8$ nanocluster (a) CR1@ $(TiO_2)_8$ (b) CR2@ $(TiO_2)_8$.

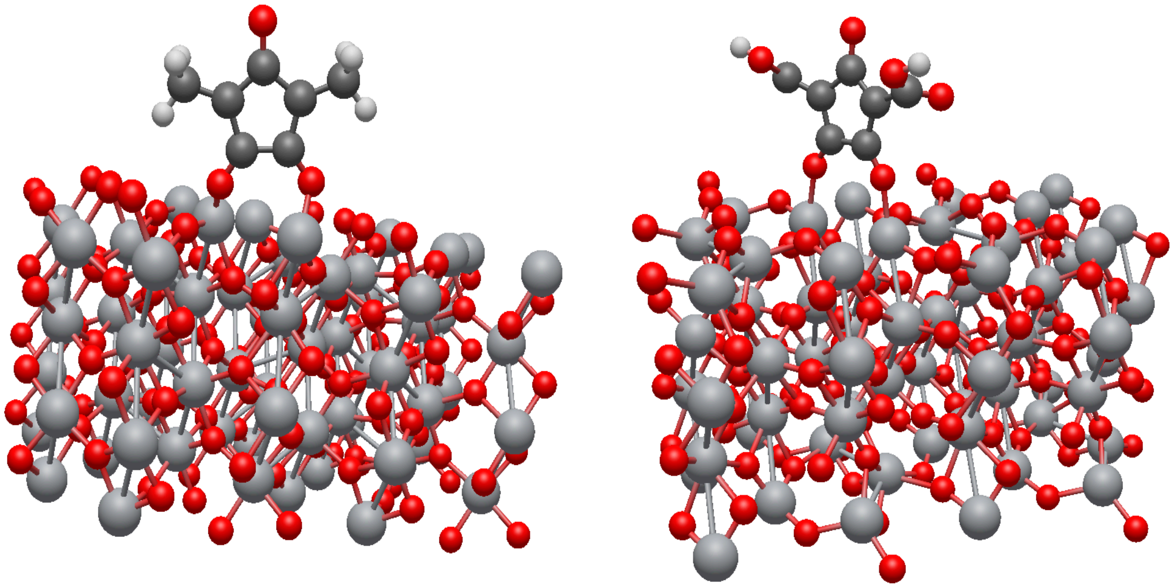

(a) **(b)**

Figure 6. Croconate dyes absorbed on $(TiO_2)_{68}$ nanoclusters (a) CR1@ $(TiO_2)_8$ (b) CR2@ $(TiO_2)_{68}$.

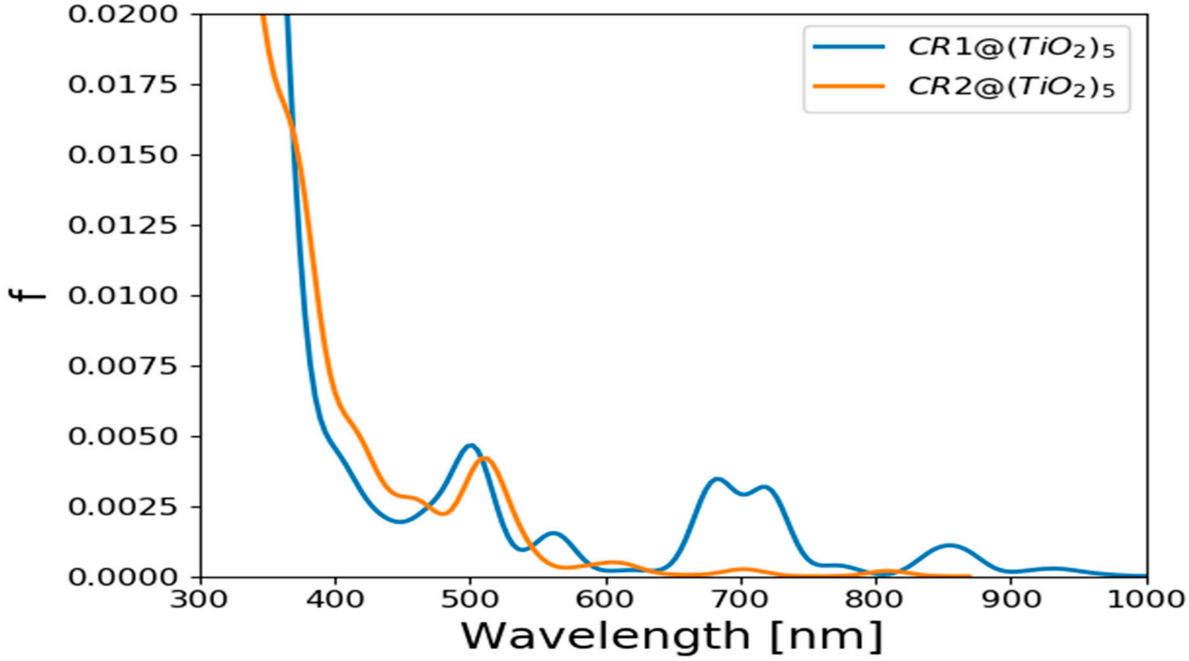

Figure 7. Expanded view of simulated UV-Vis spectrum of CR1 and CR2 absorbed on $(TiO_2)_5$ brookite cluster. The oscillator strengths were folded by Gaussians of emin=100, emax=1200nm width. The y-axis is "folded oscillator strength [1/nm].

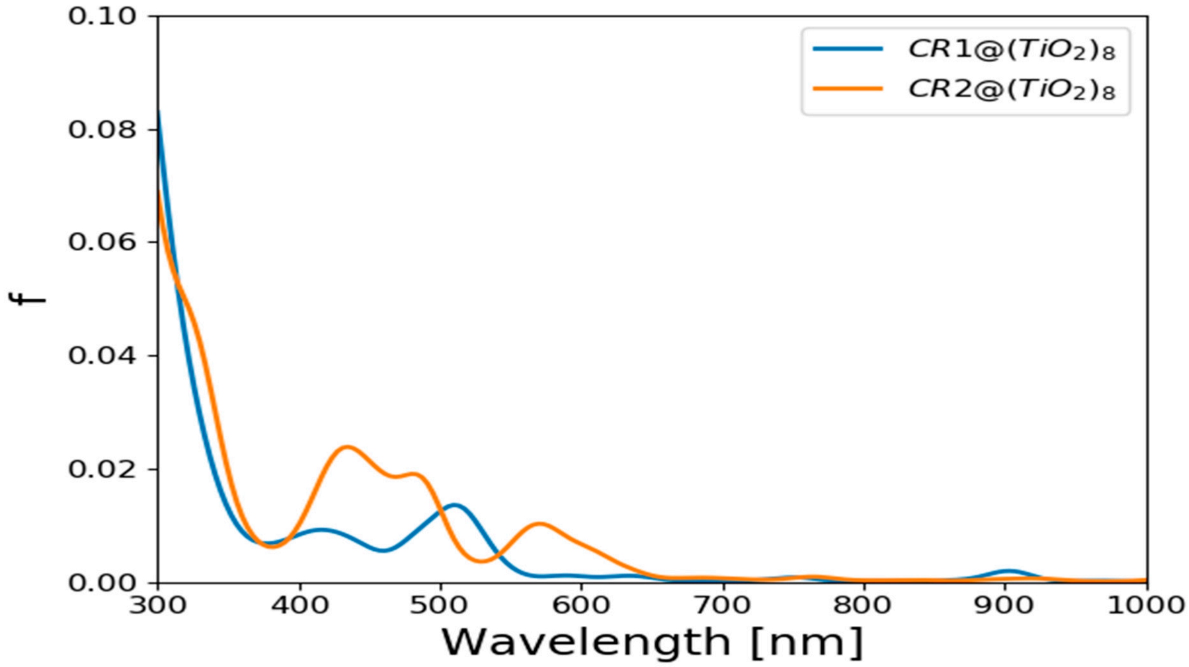

Figure 8. Expanded view of simulated UV/Vis absorption spectrum of CR1 and CR2 absorbed on $(TiO_2)_8$ brookite cluster. The oscillator strengths were folded by Gaussians of $e_{min} = 100$, $e_{max} = 1200$nm width. The y-axis is "folded oscillator strength [1/nm].
